# Community Health Nursing Education in Austria—The Need for Competences in Planning, Management and Collaboration: A Problem-Centered Qualitative Study

**DOI:** 10.3390/healthcare11243169

**Published:** 2023-12-14

**Authors:** Harald Lidauer, Harald Stummer

**Affiliations:** 1 Institute for Management and Economics in Healthcare, UMIT TIROL—Private University for Health Sciences and Health Technology, 6060 Hall in Tirol, Austria; 2 Faculty of Business Administration, Seeburg Castle University, 5201 Seekirchen am Wallersee, Austria

**Keywords:** community health nursing, primary care, competences, education and training

## Abstract

(1) Background: The Austrian health care system is extremely fragmented. Primary care is mainly provided by self-employed GPs. Other health professionals are rarely integrated into primary care. But, according to the political plans of the Austrian government, a system of community nurses and community health nurses should be implemented and several pilot projects have already been started. (2) Objective: The present study explores the skills and competences needed in the planning, management and collaboration for the change in the system and gives recommendations for community health nurse education in Austria. (3) Methodology: Fifteen qualitative, problem-centered interviews were conducted with experts in the field of community health nursing and analyzed using qualitative content analysis. (4) Results: The skills and competences often and widely mentioned are interprofessional collaboration, cooperation with other actors, systems thinking, project and change management, and basic management skills, including strategic planning, communication, accounting and finance. Areas such as health planning and lobbying are also highlighted. The main competences are broken down into subcompetences, making it possible to create a detailed competence grid. Competences in planning, management and collaboration are particularly important in the initial stages of the first implementation of a community health nursing system. (5) Conclusions: Skills and competences in these areas occupy a central position. A multilayered breakdown of these competences is required in order to create a targeted requirements profile. Due to the small-scale fragmentation of the Austrian health care system, collaboration and coordination are more difficult and costly, but all the more important. The aforementioned skills and competences represent an essential expansion of nursing education in Austria.

## 1. Introduction

The WHO considers primary care to be a central issue for the health system, as well as for the general social and the economic development of a community [1]. All in all, a wide variety of primary care concepts and models are being pursued throughout Europe and the world. While in Austria, for example, primary care is largely provided by family doctors, in countries such as Norway, Finland or Ireland, other health professionals, especially nurses, are employed to provide primary care [2]. In any case, the community health nursing model can also form a central component of a primary care concept. Improving community health is a critical factor, with guidelines [3] in place to implement it. There are numerous evidence-based recommendations to promote community participation to improve health and well-being [4]. However, when changing a system, it is important to take into account the already existing structures in the primary care sector of the country concerned.

In Austria, patients have the choice between contract physicians of the social insurance system (45%) and elective physicians (55%) [5]. There is nearly free access to individual care levels, inclusive general practitioners, specialists in private practice and hospitals. However, unlike many other countries, general practitioners do not normally fulfil a formal gatekeeping function, so specialists can also be contacted directly.

Both primary health care and specialist care in private practice are mainly provided through independently practicing private physicians; only in a very few cases are there primary health care centers established as multiprofessional and interdisciplinary units. Challenges of the Austrian health care system are the coordination of the extramural and intramural areas, on the one hand, and curative and long-term care, on the other. Past reform steps aimed, among other things, at improving coordination and cooperation within the health care system and reducing the existing fragmentation between intra- and extramural care [5]. But not even within the primary health care system has collaboration become common practice: General practitioners have only occasional meetings together with colleagues (other general practitioners), physiotherapists or home care nurses, and even more rarely with community pharmacists, social workers or midwives. They also hardly ever seek advice from specialists; at the same time, specialists do not provide joint care together with general practitioners [6].

Up to now, diploma-qualified nurses of the higher service have hardly been involved in the primary care of the Austrian population. The implementation of public health-related fields of action, such as public health nursing, community health nursing or family health nursing, as urgently recommended by the WHO [7,8], has so far been completely omitted [9]. Currently, however, there are signs of change: In its government program for the years 2020 to 2024, the Austrian federal government is initiating the “*Project Community [Health] Nurses in 500 Communities*” [10] (p. 174).

Community health nursing and public health nursing are used interchangeably by the WHO [11], whereby they define it as: “*A special field of nursing that combines the skills of nursing, public health and some phases of social assistance and functions as part of the total public health program for the promotion of health, the improvement of the conditions in the social and physical environment, rehabilitation of illness and disability*” [11] (p. 5). Public health nursing generally refers to the contribution of the individual nursing professions in the context of fulfilling public health-related tasks [12].

The model of community health nurses as a relevant profession in the primary care sector already represents an old and proven concept in countries such as Finland or Canada [13] to effectively contribute to strengthening and improving the health of people in their living environment [9]. In other countries, however, the establishment of a system of community health nursing is still in its infancy, as in German-speaking countries and in Austria, although projects—some of them limited in time—are starting to appear in some places. Until now, the Austrian health care system has been far from a nationwide or sustainable model to realize the concept of community health nursing.

At the moment, there are 116 pilot projects with approximately 180 full-time equivalents and approximately 270 people (heads) in implementation (as of June 2023) [14] in Austria, monitored by the Austrian Public Health Institute (GÖG), a daughter company of the Ministry of Health [15].

At the moment, most projects only fulfill parts of the international CHN competences (e.g., care management); however, after the evaluation of the pilot projects at the end of 2024, community health nurses are also planned [16].

Against this development, there is a central interest in exploring which skills and competences a community health nurse in Austria should have, meaning “the specific knowledge, skills and personal attributes required for a nurse practitioner to practice safely and ethically in a designated role and setting” [17] (p. 15). This will be necessary to define the levels of knowledge, abilities and skills to universities and nursing schools, but also to employers as instruments to measure job performance [18,19].

So far, there is no published study on the required skills and competences of community health nurses adapted to the Austrian health care system. Therefore, the aim of the present paper is to explore the competences needed in planning, management and collaboration for CHN in order to be successful in the fragmented Austrian health system.

## 2. Materials and Methods

### 2.1. Study Design

We used a problem-centered qualitative research design in which experts were interviewed face-to-face, according to Witzel [20,21,22]. The expert interview was used for the purpose of exploration, in order to orientate oneself in a new (thematic) field and to bring about a thematic structuring of the study area and create hypotheses [23]. The problem-centered interview combines narrative interview techniques but includes more specific guiding principles with aspects of grounded theory, especially regarding sampling techniques. For data analysis, there is no strict guideline for problem-centered interviews, but from a philosophy of science perspective, this approach is purely qualitative with as much generalizability as possible. This was performed in order to not only interpret attitudes and values, but also to provide practical normative recommendations for educational institutions and health policy.

### 2.2. Criteria for Selection of the Sampling

The following inclusion criteria were chosen for the expert interviews to be conducted as part of the qualitative study with regard to the study population:

- Experts: persons from the areas of politics, education, interest groups in health care, consulting, and operational areas with specific knowledge in the field of community health nursing (see below for a detailed listing).
- Experience: at least 3 years in the respective subject area
- Knowledge of the Austrian health care system

The selection of the persons to be interviewed (sampling) was carried out according to the method of “theoretical sampling” [24,25] as being a necessary condition in problem-centered interviews. The process involved an interactive approach that was cyclical. The steps of sampling, conducting the survey, transcription, coding and data analysis were repeated until the individual categories reached a state of theoretical saturation and the process of theory building was completed. The detailed procedure was as follows: At the beginning, the first participants were selected on the basis of a general orientation to the thematic area of the research question. The data obtained in the course of the interviews were analyzed and formed the basis for further sampling. This process was repeated until no more new information could be obtained through further interviews (theoretical saturation). In the process, the sampling procedure became increasingly specific over time [26]. The step-by-step selection of participants was conducted in particular “*to make the process orientation of the method work*” [27] (p. 219).

The method of theoretical sampling was modified in the context of the present research project in that the population was divided into five groupings and subsequently—irrespective of the question of a possible occurrence of theoretical saturation—at least two experts from each group were interviewed. The population was divided into the following five groups:

1. Politics: persons working at the Austrian Federal Ministry of Social Affairs, Health, Care and Consumer Protection, at Gesundheit Österreich GmbH or at the Austrian Chamber of Labour (who are involved in care-related issues)
2. Education: heads of study programs and lecturers in existing study programs in the field of CHN at Austrian universities and universities of applied sciences; heads and lecturers in other training and further education programs in the field of CHN at Austrian educational institutions
3. Interest groups in health care: members of interest groups in the field of primary care in Austria (e.g., Austrian Forum for Primary Care in Health Care (OEFOP), Austrian Platform for Primary Care)
4. Consulting: persons with consulting functions in the field of nursing care in Austria
5. Operational area: persons with leading activities in nursing organizations or primary care units in Austria; persons with active professional activities in connection with already existing (local) community (health) nursing projects in Austria.

The implementation of the sampling plan given in this form was successful. It should be added that some participants could have been assigned to two or more of the five groups mentioned due to their multilayered (professional) activities. In such cases, the first and second author agreed and assigned the specific interviewee to the group that most corresponded to his or her expertise.

The field access to the study population took place in such a way that the first participants were identified via the websites of institutes, authorities or companies, releases (publications, etc.), press appearances, social media appearances and Researchgate, but also by means of indirect contact via gatekeepers. Subsequently, recruitment also took place by means of the snowball principle. Contact was made electronically via e-mail.

### 2.3. Data Collection

Data collection took place in May and June 2022. Fifteen qualitative interviews were conducted in total. The average interview duration was 66 min, with the longest interview lasting 105 min and the shortest 41 min. All interviews were conducted in German.

The first contact via e-mail with a potential participant was used to give general information about the study, to enquire about the willingness to be interviewed and, in a positive case, to make an appointment for the interview itself. A few days before the agreed interview date, the respective participant was sent a prepared informative consent form, which had to be signed and returned before the interview began, and in which the digital audio recording of the interview was also agreed.

Regarding the relationship of the researchers to the participants, it should be noted that the first author, who conducted all 15 interviews, did not know any of the participants personally beforehand. The second author knew the participants of the study personally, which resulted from the fact that the community in the field of public health in Austria is very small and the persons know each other. However, the second author was not involved in the interview process.

The interviews were conducted following the guidelines (stage direction) according to Hermanns [28]. The data collection was guideline-based. The task of the interview guide was not to function as a skeleton to a structured questionnaire, but rather as an orientation framework or memory aid to thematically organize all the background knowledge of the researcher. Furthermore, the guide served to support as well as differentiate individual narrative sequences given by the interviewee [21]. Finally, the interview guide also ensured the comparability of the individual interviews [22].

The interview guide was tested and optimized in the course of test interviews. Thus, a pilot test of the interview questions was conducted with two persons who were not part of the expert group, which is why the material from these two interviews was not taken into account in the evaluation. In addition, the first three interview participants were selected in such a way that these persons could have been contacted several times if necessary in the event of problems with the interview guide or the interview questions (e.g., for a supplementary interview, queries). However, in the actual implementation of the research project, further contact with these three interview participants was not required.

The authors also pursued the objective of continuously transferring findings from interviews that had already been conducted and evaluated into the interview guide. To this purpose, for example, an evaluation of the interview guide took place after conducting and analyzing the first three interviews, in which central key informants had been interviewed. Over the course of a deliberate temporal interruption of the research process, a detailed conversation between the first and second authors was used to reflect on the results. In the end, however, the interview guide remained unchanged throughout the entire data collection phase due to a lack of need for change.

In terms of content, the interview guide referred to the main area of interest of the required skills and competences of community health nurses in Austria, with the aim of identifying focal points, establishing a connection to the Public Health Intervention Wheel [29] and the current state of opinion in the literature, as well as addressing any peculiarities/special features of the Austrian health and social system.

In this respect, the interview guide represented an overarching orientation framework with regard to the survey. In it, the problem area to be dealt with was prepared in the form of individual, thematic fields. A warm-up phase (e.g.,: “*When did you first become involved with the topic of CHN and in what form / in what context?*”, “*What is your current professional activity, how long have you been doing it and in which facets does the topic of CHN play a role?*”, “*Are you involved in any way in the establishment of the pilot projects on community nursing or in the general introduction of a system of community health nursing in Austria and if so, what is your contribution to this?*”) was followed by an open-ended introductory question, which enabled the interviewees to build up narrative sequences and to determine focal points on their own (“*In which area do you think central skills and competences lie, which a community health nurse in Austria should have in any case? … Just tell us, you can set priorities yourself—what is important to you!*”). If necessary, additional (insertion) questions followed.

In the course of the interview, more specific topics oriented to the object of the research question came into focus. The first focus was the Public Health Intervention Wheel [29] (“*Looking at the Public Health Intervention Wheel, what additional skills/competences do community health nurses have—in relation to the Austrian health care system—compared to those currently possessed by graduated diploma-qualified nurses in Austria? In which areas is additional knowledge or skills needed? Please make an assessment.*”). Another focus was on the discussion of those 13 skills or competences mentioned particularly frequently, which had been elaborated within the framework of the review in the form of the scoping review [30] and which were presented to the participants in the course of the interview (exemplary: “*Which of these skills/competences reflect, in your opinion, a central importance also for community health nurses working in the Austrian health care system? Why?*”).

Following this, the interview guide contained exit questions or concluding questions on the topic area of the required skills and competences of community health nurses in Austria (exemplary: “*Suppose you had the opportunity to design a system of CHN for Austria: Which central tasks would a community health nurse take on in this model and which skills and competences would he/she need for this?*”). In addition, the participants were given the opportunity at this point to add further representations and narrative sequences that had not yet come up in the interview up to this point.

The interviews were conducted individually via online videoconferencing and recorded. After each interview, the first author summarized field notes in a postscript (research diary). Details of the interview situation, special incidents during the interview, the behavior of the interviewer and interviewee and other conspicuous features were recorded, among other things, as an opportunity for self-reflection.

After 15 interviews, no more concepts (stories) and data with novelty value emerged; thus, it was considered that enough information had been collected to reach data saturation.

### 2.4. Data Analysis

The recorded videoconferences were transcribed verbatim by a professional transcription company, following the transcription rules of Dresing and Pehl [31]. Additionally, the transcripts were checked, corrected and pseudonymized by the first author and, after that, carefully read several times, with individual sequences of the recorded videoconferences being listened to again to once more review the transcripts. The transcripts were then uploaded to the MAXQDA Plus software (version number: MQST22-EKKPv9-ctKS2K-18fTbF-U2ubki) for content analysis [32,33]. The data analysis was carried out by means of qualitative content analysis, according to Mayring [34], whereby a combination of the techniques of summarization on the one hand and structuring on the other was chosen.

The first step of the data analysis consisted of diving into the data with a special focus on reflecting and identifying key aspects of every interview in order to answer the research issues as well as summarize key findings in context. In the subsequent phase of paraphrasing the relevant content, the individual text passages were paraphrased in a concise, descriptive form that was limited to content, whereby text elements that did not support the content (embellishments) were eliminated [34]. Once this coding was completed, the authors turned their attention to creating categories, each of which linked several codes together under a kind of umbrella. The determination and definition of the categories or the category system was carried out both deductively and inductively: A first category system was created on the basis of the results of a previously published scoping review [30] on the topic area of skills and competences of community health nurses. This first system of categories was subsequently (inductively) modified and expanded in the context of its application to the empirical material and differentiated into subcategories.

During the entire research process, the first and second authors discussed and reflected on the transcripts, the analyses, and, in particular, doubts about the assignment of codes to the individual categories in regular meetings. In addition, the categories were continuously checked and revised (formative reliability test) and at the end, a final material run was carried out on the entire material (summative reliability test). In the final step of data analysis, as proposed by Green et al. [35], we focused mainly on identifying themes.

In the present study, we investigated the central skills and competences that a community health nurse in Austria should possess. In the context of the Austrian health care system, we also investigated the additional skills and competences that community health nurses would need, based on the Public Health Intervention Wheel [25], compared to the skills and competences currently possessed by registered and qualified nurses in Austria. With reference to the previously published scoping review [30], we analyzed whether and to what extent 13 qualifications mentioned particularly frequently in international publications on the required skills and competences of community health nurses also reflect a central importance for community health nurses working in the Austrian health care system.

### 2.5. Access to the Field of Research

In the context of accessing the research field, the first author discloses that, due to several years of professional activity in the field of Austrian social insurance, he has key expertise in connection with the establishment of the Austrian health care system, the implementation of new institutions within the Austrian health care system and the (health) cost distribution among a wide variety of stakeholders. The first author is aware of these previous experiences and prejudices and considers that they should be disclosed here. It was important to put them aside in the course of the research in order to be able to address the research question at hand without bias.

### 2.6. Quality Criteria

Quality criteria serve as touchstones and targets against which the degree of scientificity of the selected method can be measured [36]. The present paper follows the following quality criteria according to Mayring [37]: process documentation, argumentative securing of interpretations, rule-governance, and proximity to the subject matter.

In the context of the methods chapter, an attempt was made to document the process of gaining scientific knowledge as precisely as possible (process documentation). The aim was and is to make the research process comprehensible for external persons (scientists, practitioners, etc.). To this end, procedures were disclosed and systematized, beginning with the compilation of the analytical instruments and ending with a detailed description of the data collection and data evaluation.

Since interpretations cannot be (objectively) verified or proven within the framework of data evaluation, the researcher must justify them argumentatively (argumentative securing of interpretations). Throughout the course of the research, care was taken to ensure an adequate prior understanding of the interpretations, thereby guaranteeing a theory-based interpretation. For discursive validation, the second author was included in the interpretation community. The individual interpretation steps and possible interpretations were discussed and analyzed between the first and second authors—if necessary several times and with theory-guided feedback.

In accordance with the quality criteria of rule-governance, a systematic processing of the underlying material took place. The analysis steps were determined in advance according to Mayring’s qualitative content analysis, followed by a subdivision of the material with the creation of meaningful units. The latter were then processed step by step and systematically with the aim of always making the research process intersubjectively comprehensible, while at the same time keeping it open for revisions and additions.

The interview participants were interviewed in their usual environment: During the videoconferences, the experts were at their normal workplace or in their normal working environment. The aim was to interview the participants in real situations (proximity to the subject matter). In addition, an attempt was made to establish as open and equal a relationship as possible between interviewer and interviewee.

### 2.7. Research Ethic Committee and Data Oversight

The Research Committee for Scientific Ethical Questions (RCSEQ) of UMIT TIROL has positively approved the study (decision of 5. 5. 2022; application number 3069). Participation was voluntary and informed consent was signed before each interview.

## 3. Results

Table 1 provides an overview of the characterization of the participants.

The study we conducted examined a broad range of skills and competences that a community health nurse in Austria should possess. The following paragraphs deal with a selection of topics from the competence area of management, as well as planning and collaboration, which emerged as particularly relevant in the interviews. The interview sequences cited in the respective context are intended to provide insights into the descriptions of the participants; they were translated from German to English by the authors.

The following competences are ranked thematically in three large groups following the number of mentions in the interviews, in the broad sections, and in the subitems.

### 3.1. Corporate Governance, Management, Human Resources

#### 3.1.1. Interprofessional Collaboration

Against the background of the clear fragmentation of the Austrian health system, the establishment of a system with community health nurses should establish a system of cooperation, especially within the system of individually-based providers as general practitioners and therapists, as well as with general hospitals, government agencies and professionals—in Austria not seen as part of the health system—like social workers and others. Therefore, “*the basic competences [of a community health nurse are] psychological, physiological, social competences*” and therefore a community health nurse is the “*health professional, who is most likely to be able and in the position to identify situations hazardous to health at an early stage*” (Int. 4, line 128–130). Beneath these competences, the self-competence to recognize the limits of one‘s own competence and then include the other network-partners seems crucial.

When establishing CHN in Austria, networking is crucial to beginning the task, a community health nurse “*has to make public her/his role and has to define the place to be the right person at the right place*” (Int. 7, line 64). Because “*we have to overcome the chimneys of the health professions*” (Int. 14, line 330) and establish a real system of interprofessional collaboration and multiprofessional teamwork.

#### 3.1.2. Cooperation with the Population and Other Actors

While the competence for interprofessional collaboration implies collaboration within the health care sector, there is also a need for competences collaborating with the population (clients, relatives, and the population in general) and with other actors (local authorities, associations and other organizations). A central precondition is the willingness for mobility and flexibility: “*I need someone who is willingly going there in person*” (Int. 6, line 461). Building up formal and informal networks and personal relations, realizing needs and conflicts and obtaining good insights into formal and informal structures of actors and the population are key. As before, this also implies a good knowledge of the Austrian political and health systems, as well as the local structures, which will be highlighted in the next paragraph.

#### 3.1.3. Systems Thinking

Connected with the ability of systems thinking is the literacy of the Austrian health system, the community-based health services and the care networks. Equipped with this knowledge, community health nurses are able to answer the following question in the best possible way for themselves and for the benefit of the community: “*Where do I place myself to create as much synergies as possible*” (Int. 3, line 310–311).

Not least because of the federalist structure, knowledge of the organization and administration of health services, but also the power structures, is crucial. Only with this knowledge, the community health nurse can point out gaps in the system. The interaction within the different elements can, within the fragmented system, save costs and lead to mediating clients and social and health services. This is related closely to having the ability to change perspectives, especially between general practitioners, social workers, politicians and relatives.

#### 3.1.4. Planning and Project Management

Nearly all interviewees highlight the importance of competences in planning and project management, both on a theoretical and a practical level. This is especially crucial when first implementing a CHN system.

Project management for a community health nurse will have a large bandwidth, from classical implementation of new health services to planning and implementing programs for individual clients, or even event management. Thereby, the classic tools of project management have to be acquired and, as in the collaboration paragraphs, be implemented, coordinating the highly fragmented health and social professionals. Additionally, project marketing or advocacy has been mentioned. Or, to sum it up, as one interviewee told us: “*As long as I do not understand project management, I can’t do or implement evidence-based practice*” (Int. 12, line 428–429).

#### 3.1.5. Strategy, Strategic Planning and Strategic Leadership

This competence comprises one main area, to recognize—based on the gaps in the actual health services—the future needs of the regional health services and the resources needed for them. A common saying is that management is about doing the right things, while strategy is about doing the right things proactively. This is then closely linked to an implementation focus with political and interprofessional competence, but not neglecting strategic leadership in case and care management. Or, as one strategy consultant puts it: The community health nurse is “*the contact person who directs, who pulls the strings, so to speak, and who has the thing under control, so to speak*” (Int. 8, line 320–321).

#### 3.1.6. Health Planning

A crucial role for a community health nurse is the role in health planning. As in systems thinking, the basis is a deep knowledge of the health system. The community health nurse will have to “*once understand: how is it organized, who manages there (…), who are the decision makers there?*” (Int. 4, line 327–328). Equally necessary is appropriate knowledge about the regional demographics and morbidities as well as a knowledge of the existing networks. Based on the existing and future needs of the population, health programs should be developed and implemented on a municipality or community level. One consultant formulates it as follows: “*So it’s really about analyzing the community well and seeing what it needs*” (Int. 5, lines 41–42).

#### 3.1.7. Management, Organization and HR including Personnel Deployment

Introducing a system of CHN also means implementing an organization. This has to be structured and managed. A community health nurse “*must be able to help build an effective organizational structure and also live by it*” (Int. 6, lines 117–118). Likewise, human resources have to be managed and—not to forget—developed. Competent health professionals have also to be deployed, which makes human resources an important competence. It is also important to keep in mind that the ethical aspect of health professionals’ human resources development is highlighted several times in the interviews.

#### 3.1.8. Change Management

Being closely related to strategy and organization, change is crucial on a structural level, but also on a community and client-centered level. Knowing the mechanism of organizational, group and individual behavior change will be important. Change management is closely related to project management, but goes beyond. While project management is knowing the tools of implementing or organizing, change management is seen as complementary meta-competence to project management. The specific difficulties of change management are the complexity of and the concern of actors in the health system. So, empathically, sensitive and sustainable change processes have to be stimulated. One interviewee formulates it as follows: “*We have the focus practically on the medical care and there it is simply important in an efficient change management to really (…) slowly generate this awareness, what the role of the nurse is and what added value (…) this role can also bring*” (Int. 4, line 341–346).

### 3.2. Communication and Lobbying

#### 3.2.1. Communication

Communication is closely linked with Interprofessional Collaboration and Cooperation with the Population and Other Actors, but has been mentioned as specific competence within the interviews. There, communication skills are related but different to collaboration and cooperation. A participant from the operational area states: “*The communication issue is a very important thing. Because you have to communicate within the system*” (Int. 2, lines 258–259). Other respondents note that the community health nurse must be able to “*reach out to people and so must be able to build trust communicatively*” (Int. 7, lines 57–58), at the same time he or she must also be able to “*stand up in front of a community council and speak eloquently*” (Int. 3, line 466–467). Communication also relates to the next part, advocacy and especially lobbying.

#### 3.2.2. Lobbying

Population health has to be communicated but also lobbied for. This includes classic marketing channels like social media, but also political lobbying, convincing relevant stakeholders and others and advocating the community and community health. The community health nurse must thus be able to “*act at the policy level*” (Int. 10, line 40) and “*act as an advocate for all persons and target groups for whom she is responsible*” (Int. 14, line 87–88).

### 3.3. Accounting and Finance

According to the participants, this competence comprises different fields of areas. Starting from budgeting, it also consists of understanding costs of intervention including the identifying of potential cost savings. Resource management is another field that was strongly addressed by respondents. Which resources exist in a community-based setting and which interventions can be set there? The resource management is in most cases connected to a financial decision that has to be related to legislation (e.g., drugs and other remedies).

### 3.4. Summary of the Findings

Table 2 summarizes the findings and provides an overview of the results with the generalizations of the interviews.

## 4. Discussion

The results of the research show that skills and competences in the area of planning, management and collaboration occupy a central and indispensable point in the overall concept of competences (competence grid) of a community health nurse. They are even more important in the initial phase of the first implementation of a CHN system: here, skills, such as interprofessional collaboration, cooperation with the population, planning and project management, management, organization and human resources, lobbying, and change management, form a distinctive interface that can contribute to the success of such an implementation, and are, thus, also important for a community health nurse working in Austria.

Our qualitative study has shown that it is not sufficient to demand general planning competences, general management competences or general competences of collaboration. Rather, a multilayered and wide-ranging differentiation and breakdown of these competences is required in order to create a targeted profile of requirements for a community health nurse, but also to design their training or the contents of this training in a needs-oriented and practical way. Therewithin, systems competence, change management and personal competence seem to be crucial as future skills [11,38,39]. In this respect, our competence grid with the listed main competences and the numerous subcompetences reflects a degree of detail that corresponds on the one hand to the relevant explanations of the experts, and, on the other hand, forms a practical framework for the implementation of a concept of CHN in Austria.

The competences and subcompetences in the area of planning, management and collaboration presented in the results section are close to those mentioned in international publications on the required skills and competences of community health nurses; however, they go beyond to provide details of the existing competence profiles [40,41], the Public Health Intervention Wheel [29] or reviews of competence [30], as these regularly mention only competences but do not break them down into subcompetences.

A special feature of the Austrian health care system is that there is a clear fragmentation. Compared internationally, primary care is nearly exclusively provided by self-employed individual medical practices [5]. This small-scale fragmentation affects almost all players and health care professions within the health care system. As a result, collaboration, cooperation and coordination between the individual players is more difficult and costly, but all the more important. For this reason, targeted competences in the area of planning, management and collaboration of the community health nurse, but also of all other health care professions, form a decisive basis and prerequisite for a functioning and efficient health care system.

In Austria, both primary health care and specialized care in private practice are currently provided mainly by independently practicing physicians [5,30]. With the exception of the medical profession, the individual professional groups within the health care system have relatively little responsibility in an international comparison. The goal is to reconsider this centering on the professional group of physicians—if only for the reason of the existing shortage of physicians, which will become even more apparent in the future—and to subject it to an intensified evaluation and change process. This must also be accompanied by a revision of professional rights in legislation [9], which—in line with international models—must result in an expansion of the professional rights of other, non-physician occupational groups within the health care system, such as the community health nurse. Therefore, expanding the status of community health nurses and taking responsibility for processes will change the power distribution within the health system. Introducing concepts like collaborative leadership [42] highlights the necessity for strong literacy in these competences.

For the education of a community health nurse working in Austria in the future, the results of the present research mean that skills and competences in the field of planning, management and collaboration have to be included in the corresponding curricula in a targeted and practice-oriented way. A demand-oriented and differentiated education should be developed, promoted and supported explicitly for this new professional group. It is important to refrain from mere general subject bundles, but rather to pursue the goal of explicitly defining the individual main areas of competence as well as subcompetences in the most efficient, detailed and comprehensive form possible in the legal framework for studies.

In this context, it seems to make sense—building on a broad and general nursing education at the bachelor level—to integrate the mentioned areas of competence within the framework of a master’s program or at the master’s level. In accordance with the research results, this should be explicitly coordinated with the framework conditions and needs of the Austrian health care system. The research results show that education and training must be based on a secure theoretical foundation, but at the same time, practical knowledge (practical elements) in the competence area of planning, management and collaboration are indispensable [30,43]. But as mentioned, especially the implementation needs special skills in personal mastery, systems thinking and change management [38]. From this perspective, it seems advisable to focus on practical elements within the framework of the master’s degree education, be it through corresponding professional internships, teaching staff from practice or forms of cooperation according to the buddy principle. This idea would also correspond to a—possibly optional—variant of an in-service training. Key recommendations for community health nurse education and training should also consider and incorporate the content of the NICE guidelines [3].

### Limitations

Methodological limitations result from the selection of cases (participants). A reduction of these limitations should be achieved by the use of the principle of theoretical saturation as well as by the fact that the population was subdivided into several groupings and—independent of the question of a possible occurrence of theoretical saturation—at least two experts from each grouping were interviewed. But, as for being a new area within the Austrian health care sector, the number of potential interview partners having expertise in this field was very limited. We cannot rule out that during the ongoing implementation process, the competences will change during the next years and with the amount of growing expertise, more detailed information will exist. Nevertheless, as a starting point for an educational reform, they should be more than sufficient.

Limitations in the transferability of the study results to other countries result from the fact that the health and social systems, as well as the legal systems differ. Therefore, further research comparing CHN educational needs on a country-basis is required.

Lastly, it is also important to emphasize that this article deals exclusively with skills and competences of the community health nurse in the areas of planning, management and collaboration. In addition, however, there are other central and equally important competence areas of the community health nurse that could not be addressed here. In particular, generic competences, public health competences, or health and care management competences in combination with advanced clinical skills should be mentioned.

## 5. Conclusions

Skills and competences in planning, management and collaboration occupy a central position in the competence grid of community health nurses. A multilayered and far-reaching differentiation and breakdown of these competences is required in order to create a targeted requirements profile. Due to the small-scale fragmentation of the Austrian health care system, collaboration, cooperation and coordination between the individual actors is more difficult and costly, but all the more important. The aforementioned skills and competences represent an essential expansion of nurses‘ education in Austria. Future research should address the question of which criteria the education and training of a community health nurse in Austria has to fulfill in order to reflect the skills and competences defined in this study and which requirements a corresponding curriculum has to meet. In addition, there is a need to focus on other key bundles of competences, such as generic competences in particular, public health competences, and health and care management competences in combination with advanced clinical skills.

## Figures and Tables

**Table 1:** Overview of the characterization of the participants.

Participant	Sex	Age	Group
1	female	older than 50 years	group 2—education
2	male	older than 50 years	group 5—operational area
3	male	older than 50 years	group 3—interest groups health care
4	female	older than 50 years	group 2—education
5	female	older than 50 years	group 4—consulting
6	male	younger than 50 years	group 5—operational area
7	male	older than 50 years	group 5—operational area
8	male	older than 50 years	group 4—consulting
9	female	younger than 50 years	group 1—politics
10	female	younger than 50 years	group 5—operational area
11	female	younger than 50 years	group 1—politics
12	female	younger than 50 years	group 2—education
13	female	younger than 50 years	group 2—education
14	female	older than 50 years	group 1—politics
15	male	older than 50 years	group 3—interest groups health care

**Table 2:** Summary of competences and subcompetences.

Competence	Subcompetences
Interprofessional Collaboration	interdisciplinary networking; interface management; coordination; cooperation (also with professional groups outside the health system, e.g., social workers, debt counselling); administrative competence; linkage of systems; recognizing the limits of one’s own competence; multiprofessional teamwork
Cooperation with the Population and Other Actors	willingness for local mobility and flexibility; willingness to participate in the public life of the community; actively approaching other people; relationship building; identification of problem areas, gaps, supply deficits; conflict management and ability to reach consensus; articulation and representation of own positions (standing); recognition of people’s own competences; interface management with actors outside the health and social system; knowledge of the Austrian health system and the community-based care system; ability to gain insight into formal and informal structures of actors and the population
Systems Thinking	knowledge of the Austrian health care system, the community-based care system as well as existing care networks; knowledge about interaction of the care systems; knowledge of decision-makers and federal (power) structures in Austria; carrying out cost-benefit assessments in connection with interventions; ability to change perspectives
Planning and Project Management	theoretical knowledge in planning and project management; practical experience in project work; setting up and executing an efficient organizational structure; organizing events; scheduling and distributing work packages; coordinating staff and support staff; conducting data collection and project evaluations; self-management and time management; communicating with decision-makers and advocating with them on a project-related basis
Strategy, Strategic Planning and Strategic Leadership	strategic care planning; ability to identify gaps and derive needs from them; optimization of existing systems and efficient integration of these systems into the care structure; participation in (political) strategies within the framework of higher-level, cross-community processes; strategic and planning skills related to interface management and setting up of care services; strategic and planning skills related to demand planning at individual level
Health Planning	knowledge of the Austrian health care system, the community-based care system as well as existing care networks; general knowledge of the region in which the community health nurse is working and of the local (population) structure; analysis of care systems in the municipality; identification of problem areas and evaluation of resulting needs (e.g., through assessments); original planning of the supply system in the municipality and coordination with the local system partners in this regard; participation in higher-level, cross-municipality processes and strategies
Management, Organization and HR including Personnel Deployment	establishment of an efficient organizational structure, EDP organization as well as office organization; network competence; interface management; basic knowledge in the field of human resources and personnel development; deployment of competent health professionals; ethical and effective management (ethical reflection, economic ethical considerations)
Change Management	ability to bring the role and function of the community health nurse more into the focus of the Austrian population; ability to anchor the approach of prevention and health promotion more strongly within the population; ability to position the topic of health and how to deal with it in the population much earlier, starting with kindergarten and elementary school age; consideration and analysis of attitudes, levels of knowledge, behaviors in the community in this regard; knowledge of mechanisms of organizational, group and individual behavior change; stimulation of empathetic, sensitive and sustainable processes of (change) in the community in this regard
Communication	interprofessional communication; communication with clients and relatives; communication with stakeholders
Lobbying	community development and advocacy; acting on political level; advocacy role; knowledge of Austrian political structures (decision makers, procedures etc.); knowledge of (regional) political communication forms and channels; public relations; social marketing
Accounting and Finance	general knowledge of budgeting and financial management; ability to take an economic view of the community (performance possibilities, possible interventions, cost savings); optimization of existing resources for best possible outcome (resource management, elaboration of related solutions); initiation of political intervention processes for allocation of additional (financial) resources; knowledge of the connection between financial decisions and legislation

## Data Availability

Due to privacy regulations, the interview transcripts are not public available. The MAXQDA codes can be requested from the authors.
